# Effect of Iron Phase on Calcination and Properties of Barium Calcium Sulfoaluminate Cement

**DOI:** 10.3390/ma14195813

**Published:** 2021-10-04

**Authors:** Jun Chang, Jixin Zhang, Yanchen Yuan, Kai Cui

**Affiliations:** 1 School of Civil Engineering, Dalian University of Technology, Dalian 116024, China; mlchang@dlut.edu.cn (J.C.); ZJXin@mail.dlut.edu.cn (J.Z.); 2 School of Materials Science and Engineering, University of Jinan, Jinan 250022, China

**Keywords:** barium calcium sulfoaluminate cement, iron phase content, hydration

## Abstract

In this paper, the effect of iron phase content on the calcination and properties of clinker and barium calcium sulfoaluminate cement was studied. The compressive strength of the samples was tested and combined with an XRD and SEM-EDS analysis, and the microstructure and composition of the barium calcium sulfoaluminate clinker and hydrated samples were characterized. The results showed that the oval-shaped particles were C_2_S minerals, and the hexagonal plate-shaped or rhombohedral dodecahedral particles were C_2.75_B_1.25_A_3_$\bar{S}$. The Ba element was mainly distributed in the barium calcium sulfoaluminate region, and some of it was dissolved in C_2_S; the Fe element was distributed between C_2.75_B_1.25_A_3_$\bar{S}$ and C_2_S crystal grains in the form of an iron phase solid solution, which acted as a solvent. When the iron phase composition was C_4_AF and the iron phase content was 5%, the early hydration and later strength were better, and the compressive strength after curing for 1, 3 and 28 days was 73.2 MPa, 97.9 MPa and 106.9 MPa, respectively. A proper amount of the iron phase can reduce the eutectic point of the sintered mature material system, increase the amount of liquid phase, reduce the viscosity of the liquid phase, effectively accelerate the migration of mineral ions and promote the formation and growth of minerals.

## 1. Introduction

The main minerals of calcium barium sulfoaluminate cement are C_2.75_Ba_1.25_$\bar{S}$ and β-C_2_S. It has the advantages of fast hardening, early strength, anti-corrosion and corrosion resistance, and it plays an important role in special engineering applications [1,2,3,4,5,6]. In addition, many researchers recognized that the iron phase in cement was a solid solution series with a composition in the range of C_2_F–C_6_A_2_F. Generally, C_2_F, C_6_AF_2_, C4AF and C_6_A_2_F are the representative minerals of this series. It has some influence on the cement reactivity. In Portland cement, C4AF and C_3_A melt into a liquid phase during the calcination process, which can promote the formation of C_3_S [7]. Zhu Ming et al. used chemical reagent ingredients to research the formation process and hydration performance of the undoped iron phases C_2_F, C_6_AF_2_, C_4_AF and C_6_A_2_F. The results showed that the iron phase always diffused and replaced the C_2_F lattice through Al^+3^ ions. When the different components were not doped, the synthesis of the iron phase became difficult with the increase of the Al/Fe ratio. The iron phase represented by C_4_AF not only has good wear resistance and corrosion resistance, but also has good mechanical properties [8]. Wang Yanmou et al. studied the formation mechanism of the iron phase in ferro-aluminate cement and clarified the formation mechanism of the four representative minerals C_2_F, C_6_AF_2_, C_4_AF and C_6_A_2_F [9].

On this basis, the formation process of the iron phase in ferro-aluminate cement clinker was studied. The results showed that the composition of the iron phase was close to C_6_AF_2_; the formation of the iron phase was due to the Al^3+^ into the C_2_F lattice. The final composition of the iron phase depended on the Al/Fe atomic weight ratio of the raw material and the sintering temperature [10]. Huang Yeping et al. used XRD, TG and other test methods to study the influence of Fe_2_O_3_ on the formation of C_4_A_3_$\bar{S}$. Studies have shown that the addition of Fe_2_O_3_ can promote the absorption of f-CaO in the clinker, increase the amount of the liquid phase and improve the burnability of C_4_A_3_$\bar{S}$ [11]. Li Yanjun et al. studied the influence of Fe_2_O_3_ on the formation of alite-sulfoaluminate cement clinker minerals with pure chemical reagent ingredients. The results showed that when calcined at a low temperature, Fe_2_O_3_ was not conducive to the absorption of f-CaO in the system; a certain amount of Fe_2_O_3_ can promote the formation of C_3_S and C_4_A_3_$\bar{S}$ at a high temperature, which was conducive to their coexistence in the clinker. Furthermore, when the content of Fe_2_O_3_ was higher, it would hinder the formation of C_3_S and reduce the content of C_4_A_3_$\bar{S}$ [12].

Previous studies on sulfoaluminate cement have shown that the iron phase played an important role in the firing process of sulfoaluminate cement, which has attracted more and more attention. A lot of research has been conducted on sulfoaluminate cement [13,14,15,16,17]. However, there are few studies on the influence of iron phase content on the calcination and performance of barium calcium sulfoaluminate cement. Therefore, it is of great significance to study the iron phase in barium calcium sulfoaluminate cement and obtain the law and understanding of the influence of iron phase content on barium calcium sulfoaluminate cement. The research results have important theoretical value for enriching and improving the barium calcium sulfoaluminate cement system, have important guiding and practical significance for the selection of raw materials and production practices and provide a reasonable and effective method for studying the iron phase [18,19,20,21].

## 2. Experiment

### 2.1. Materials

The analytical pure chemical reagents including CaCO_3_, BaCO_3_, Al_2_O_3_, SiO_2_, Fe_2_O_3_ and CaSO_4_·2H_2_O were used in the experiment, produced by Tianjin Guangcheng Chemical Reagent Co., Ltd, Tianjin, China. The mineral composition of the designed clinker was C_2.75_B_1.25_A_3_$\bar{S}$, C_2_S and the iron phase [22,23,24,25]. The composition of each group is shown in Table 1. The weighed raw meal was mixed with water and put into the steel tank of the planetary mill for stirring, then the mixed raw material was put into a blast box for drying. Finally, the raw material was pressed into a 60 mm × 10 mm test cake and put it into a blast drying box to keep it warm for 1 h. The samples were put into a high-temperature furnace for calcination, the temperature was raised to 1350 °C with a heating rate of 5 °C/min and kept warm for 2 h and then the samples were taken out and cooled to room temperature quickly [26,27,28,29,30,31].

### 2.2. Experimental Methods

The calcined cement clinker was grounded and the fineness was less than 200 mesh sieves, and the sieve residue was within the range of 1–5% (74 μm sieve opening). Then, the grounded cement clinker was mixed with water (the water to cement ratio was 0.3) stirred and vibrated for 3 min, and paste was poured into a mold (2 × 2 × 2 cm^3^) and placed in a standard curing at a room temperature of 20 °C for 1, 3 and 28 days. Finally, the mechanical properties of cement were measured after 1 day, 3 day and 28 days of curing ages according to standard ISO 679:2009, the 6 samples were tested for per age, the error bars were calculated as the standard deviation and the composition structure was analyzed. The compressive strength of each sample was carried out with a loading rate of 2400 N/s ± 200 N/s.

The QM-4L planetary ball mill (Nanjing Nanda Instrument Factory, Nanjing, China) was used, the ZB101-1 electric blast box (Zibo Instrument Factory, Zibo, China) was used and the box-type resistance furnace (Longkou Electric Furnace Factory, Yantai, China) was used. A WDW-200E microcomputer-controlled electronic type universal testing machine (Jinan Testing Machine Factory, Jinan, China) with a loading rate of 60 N/s and an X-ray diffractometer (XRF, Shimadzu, Kyoto, Japan) were used to detect the composition of the cement, and XRD (XRD, Brooke, Germany) measurements were performed using a D8 Advance X-ray diffractometer with Cu target (Cu Kα_1,2_ radiation). The working voltage and working current are 40 kV and 40 mA, respectively, the test range is 5–80° (2θ). The step size is 0.02° with one step of 0.1 s. A Nova Nano SEM 450 scanning electron microscope (SEM, FEI, Hillsboro, OR, USA) was used to observe the morphology of the cement. The sample was sprayed with gold for 60 s, the pressure was 30 Pa, the current was 15 mA, the electron beam spot was 3 and the acceleration voltage was 3 kV.

## 3. Results and Analysis

### 3.1. Effect of C_4_AF Content on Barium Calcium Sulfoaluminate Cement

#### 3.1.1. Mechanical Properties

**Figure 1:**
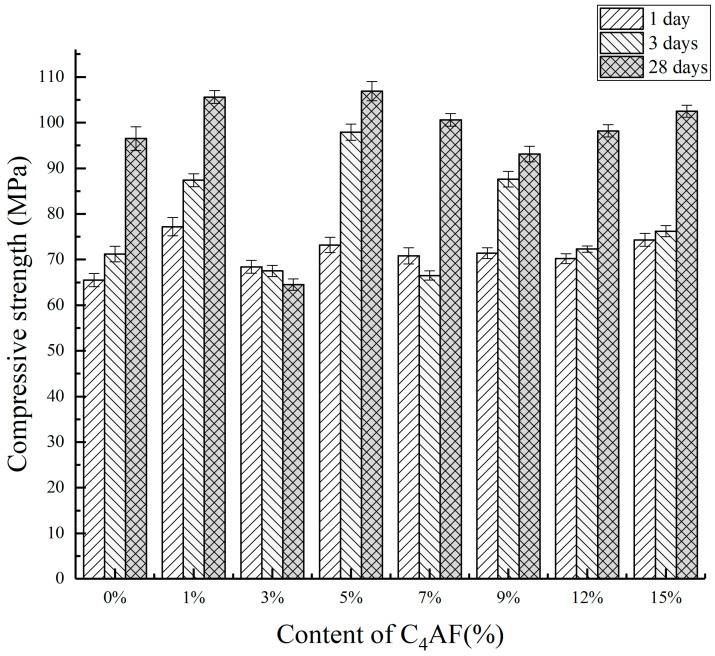
Compressive strength of the different C_4_AF content of the clinker at 1350 °C.

It can be seen from Table 2 and Figure 1 that when the iron phase content was lower than 1%, the compressive strength was higher. This was due to the high content of C_2.75_B_1.25_A_3_$\bar{S}$ and C_2_S in the mineral composition of the clinker calcination at 1350 °C. The hydration of barium calcium sulfoaluminate provided early strength, and the hydration of C_2_S mainly provided later strength. Although it could provide higher strength when the iron phase content was relatively low, in practice, when the iron phase content was low, there were fewer flux minerals during the formation of minerals, which was not conducive to the migration of ions in the minerals during the clinker calcination process.

With the increase of the iron phase, the compressive showed a decreased trend, but when the content of the iron phase reached 15%, the early strength and later strength had an improvement, and the compressive strength of 3 days and 28 days were 76.2 MPa and 102.5 MPa, respectively. However, part of the samples melted, which may bring certain difficulties to the kiln operation in the actual production. A proper content of the iron phase can reduce the calcination temperature, reduce the viscosity of the liquid phase, increase the amount of the liquid phase and promote the formation of minerals. When the content of the iron phase was 5%, the compressive strength was optimal. The compressive strength of 1, 3 and 28 days were 73.2 MPa, 97.9 MPa and 106.9 MPa, respectively.

#### 3.1.2. XRD Analysis

**Figure 2:**
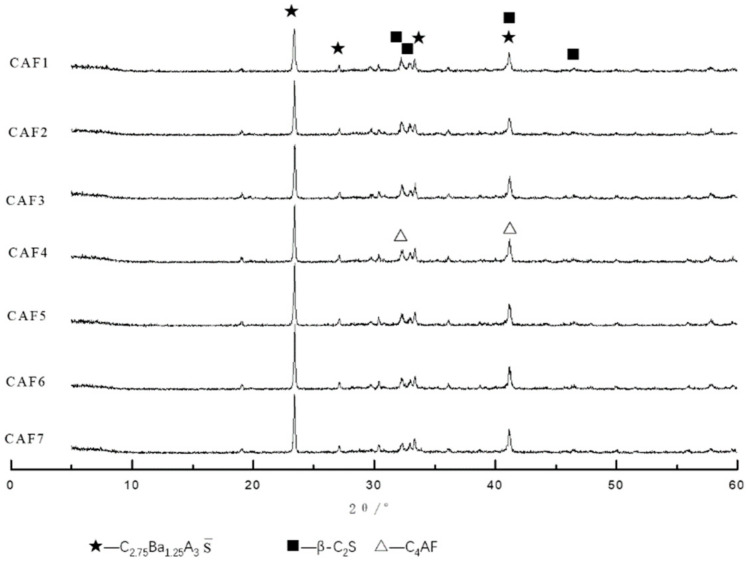
XRD patterns of different clinkers at 1350 °C.

It can be seen from Figure 2 that the main minerals of the calcium barium sulfoaluminate cement clinker was C_2.75_B_1.25_A_3_$\bar{S}$ and β-C_2_S, and there were fewer impurity peaks and the diffraction peak was sharp, indicating that the mineral crystals were well developed. It can be found that the intensity of the diffraction peaks gradually increased with the increase of the C_4_AF content: when the content of C_4_AF was 5%, the first and second diffraction peaks of C_2.75_B_1.25_A_3_$\bar{S}$ were sharper and the diffraction peak intensity was higher. This showed that there were more of C_2.75_B_1.25_A_3_$\bar{S}$ formed in the clinker, and the crystal crystallization condition was good. The diffraction peak of C_4_AF was not obvious due to the diffraction peak of C_4_AF and the diffraction peak of C_2_S was partially overlapped.

#### 3.1.3. SEM Observation

**Figure 3:**
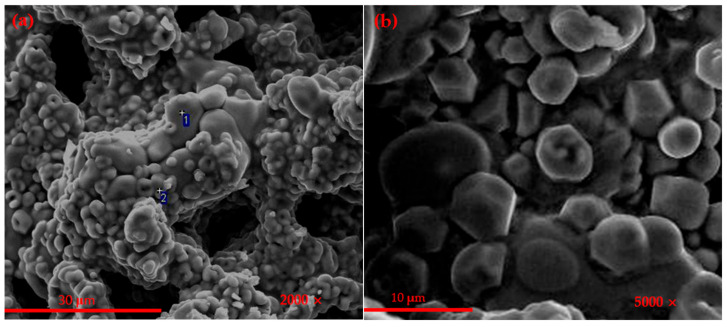

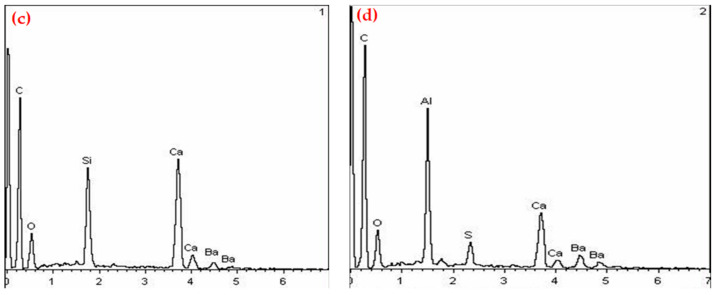
SEM-EDS micrographs of (**a**,**b**) CAF_1_ clinker, (**c**) 1-EDS, (**d**) 2-EDS.

Figure 3 is the SEM-EDS of the barium calcium sulfoaluminate cement clinker with 1% C_4_AF content. It can be seen that the clinker had less liquid phase, the grain boundaries between the particles were blurred and the structure was looser. Combined with the EDS at Figure 3c, it can be concluded that the oval granular mineral at this point was β-C_2_S, and the crystal grain size was about 10 μm. Combined with the EDS at Figure 3d, a large number of small rhombohedral particles can be observed. These particles were C_2.75_B_1.25_A_3_$\bar{S}$, and the grain size was about 3–5 μm. Because the liquid phase was lower in the sintering process, it was not conducive to the migration of mineral ions during the clinker calcination process, which would affect the formation and development [29,31].

**Figure 4:**
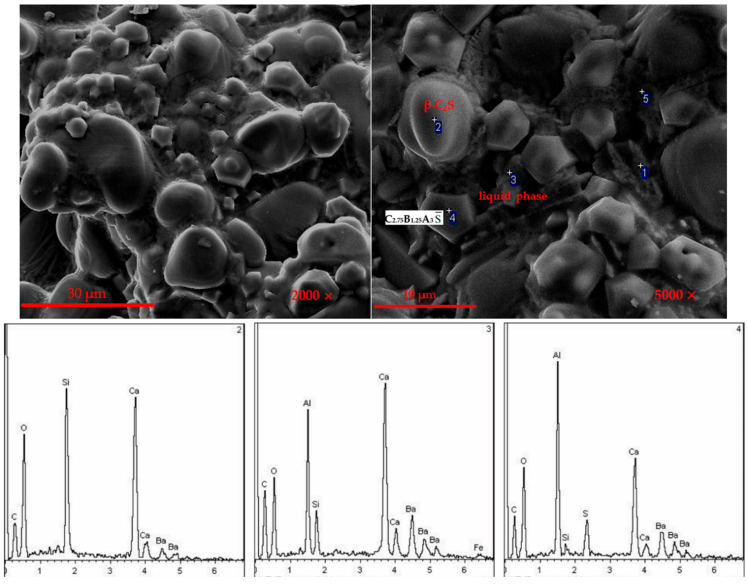
SEM-EDS photographs of CAF_3_ clinker.

Figure 4 is the SEM-EDS of the barium calcium sulfoaluminate cement clinker with 5% C_4_AF content. It can be seen that the minerals were in an aggregated state, with a certain liquid phase and the grain boundaries between particles were clearer. The crystal size was ranging from 10 to 20 μm. Combined with the SEM-EDS of 2-point, the mineral was β-C_2_S. There were a large number of small hexagonal particles, with a crystal size of 3–5 μm. Combined with the SEM-EDS of 4-point, the mineral was C2_.75_B_1.25_A_3_$\bar{S}$. Part of the irregular melting phase can be seen at the interface of these two minerals. Combined with the SEM-EDS of 3-point, it was a liquid phase mineral containing iron.

**Figure 5:**
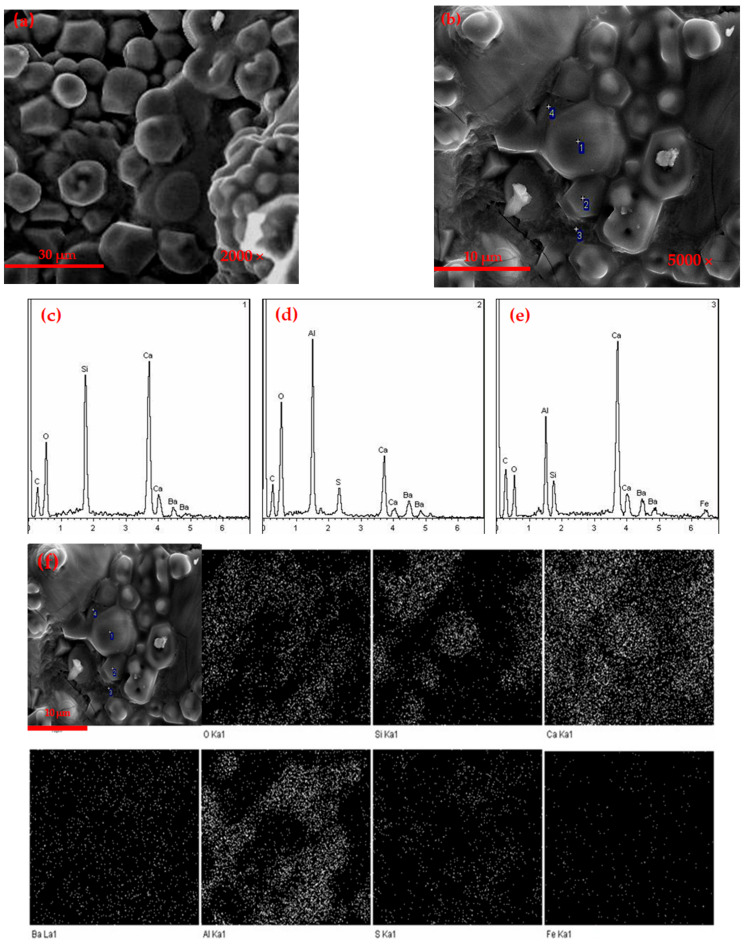
SEM-EDS photographs of (**a**,**b**) CAF5 clinker, (**c**) 1-EDS, (**d**) 2-EDS, (**e**) 3-EDS, (**f**) scanning photograph of the cement clinker CAF5.

Figure 5 is the SEM-EDS of the barium calcium sulfoaluminate cement clinker with 9% C_4_AF content. It can be seen that the mineral structure was loose, the grain boundary was not clear and the liquid phase was higher than CAF1 and CAF3. Combining the EDS at 1, 2, and 3-points, it can be seen that the egg-shaped particles were β-C_2_S, and the hexagonal plate-shaped mineral particles were C_2.75_B_1.25_A_3_$\bar{S}$. It can be seen that some of the hexagonal flake-shaped particles were irregular morphology. This was because the iron phase content was high, and when the calcination temperature was 1350 °C, the eutectic point of the mineral was lower, which made the barium calcium sulfoaluminate mineral partially dissolved.

The Ba element was mainly distributed in the hexagonal plate-shaped barium calcium sulfoaluminate mineral area, the Ba^2+^ replaced Ca^2+^ and formed barium calcium sulfoaluminate mineral; however, there was also a part of the Ba element distributed in the egg-shaped dicalcium silicate area. Ba^2+^ were solid-dissolved into the C_2_S minerals, which can effectively activate the crystal lattice and increase the activity of C_2_S, which was one of the reasons for the higher strength of this series of cements. Fe elements were distributed throughout the viewing area. During the formation of minerals, iron-aluminum phases were formed, and iron phases were distributed in the minerals between the crystal grains of C_2.75_B_1.25_A_3_$\bar{S}$ and C_2_S. At the same time, some iron may be dissolved in the C_2.75_B_1.25_A_3_$\bar{S}$ and C_2_S minerals.

### 3.2. XRD and SEM Analysis of Hydration Samples

#### 3.2.1. XRD Analysis of Hydrated Samples

**Figure 6:**
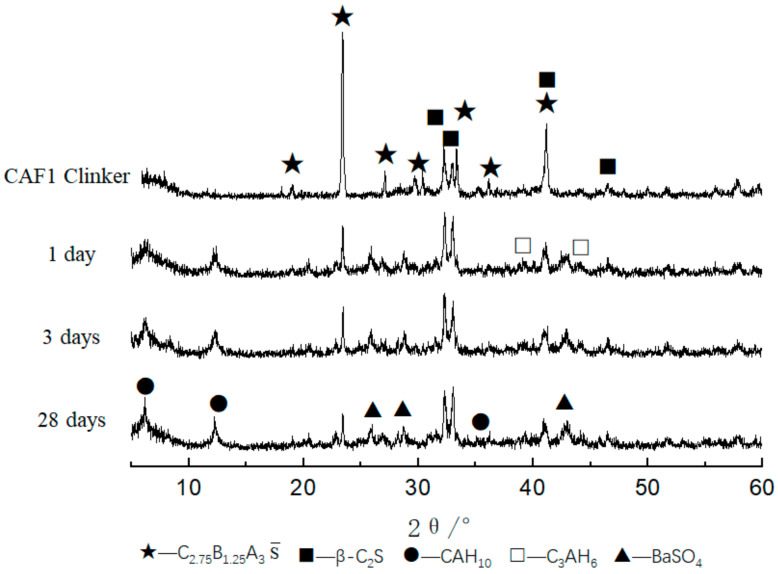
XRD patterns of CAF1 at different curing times.

**Figure 7:**
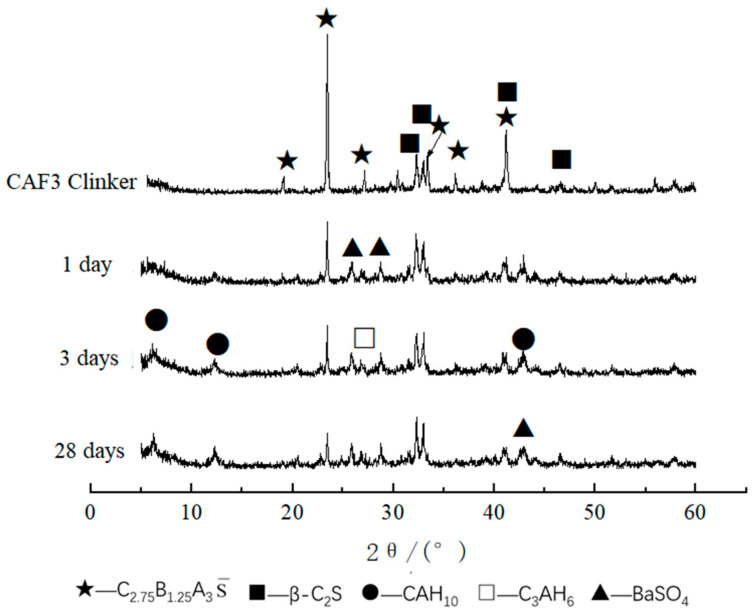
XRD patterns of CAF3 at different curing times.

**Figure 8:**
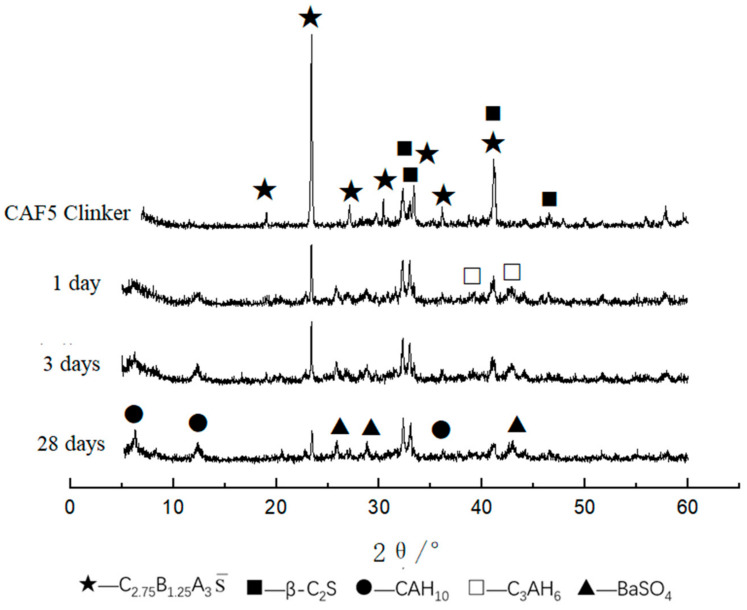
XRD patterns of CAF5 at different curing times.

The XRD patterns of CAF1, CAF3, and CAF5 at different curing times are shown in Figure 6, Figure 7 and Figure 8. It can be seen from Figure 6 that compared to the diffraction peaks of the cement clinker, the diffraction peaks of the barium calcium sulfoaluminate minerals were reduced, indicating that the early hydration rate of barium calcium sulfoaluminate minerals was very fast, which provided early strength. Comparing the XRD pattern of 3 days and 28 days hydration products, there was basically no obvious change, indicating that the hydration degree of minerals at 3 days was already very high, and the hydration was basically completed at 28 days. C_2_S had a small amount of hydration, the early hydration speed was relatively slow, and the diffraction peaks were not changed obviously along with the curing time. This feature provided a guarantee for the later strength growth of this kind of cement. The diffraction peaks of CAH_10_ gradually increased along with the curing time. The main hydration products were BaSO_4_, CAH_10_ and a small amount of C_3_AH_6_, and there were also some C_2_S and C_2.75_Ba_1.25_A_3_$\bar{S}$ that had not been hydrated.

Figure 7 is the XRD pattern of the CAF3 (5%) clinker and hydrated samples at different curing times. It can be seen that the main hydration products were BaSO_4_, CAH_10_ and C_3_AH_6_. Compared with the XRD patterns of the clinker and the hydration products at 1, 3 and 28 days, it can be seen that the XRD pattern of the CAF3 hydrated sample was similar to that of CAF1.

Figure 8 is the XRD pattern of the CAF5 (9%) clinker and hydrated samples at different curing ages. It can be seen that the main hydration products were BaSO_4_, CAH_10_ and C_3_AH_6_, and the diffraction peaks of CAH_10_ increased along with the curing time. Compared with the XRD patterns of the clinker and hydrated samples for 1, 3 and 28 days, it can be seen that the XRD patterns of the CAF5 hydrated samples were similar to CAF1 and CAF3.

**Figure 9:**
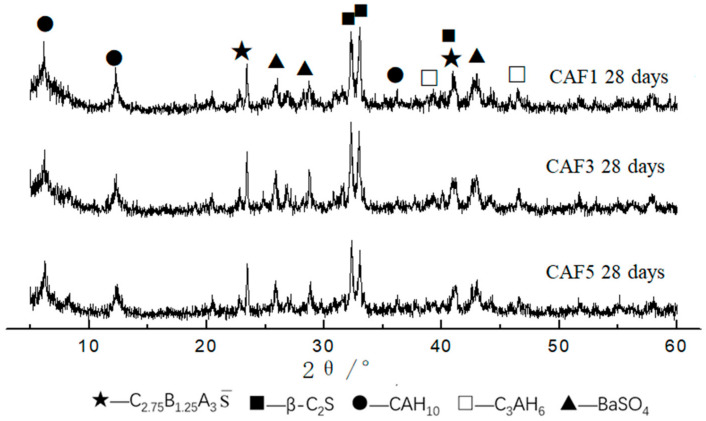
XRD patterns of different hydrated samples at 28 days.

Figure 9 is the XRD patterns of CAF1, CAF3 and CAF5 at curing for 28 d. It can be seen that the main hydration products of the samples were BaSO_4_, CAH_10_ and unhydrated C_2_S and C_2.75_B_1.25_A_3_$\bar{S}$. When CAF1 was curing for 28 days, the peak value of CAH_10_ in the hydrated product was higher than the other two. This was also one of the reasons for the higher strength of CAF1.

#### 3.2.2. SEM Analysis of Hydration Samples

**Figure 10:**
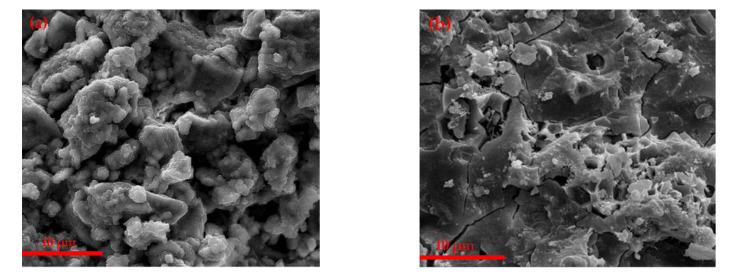

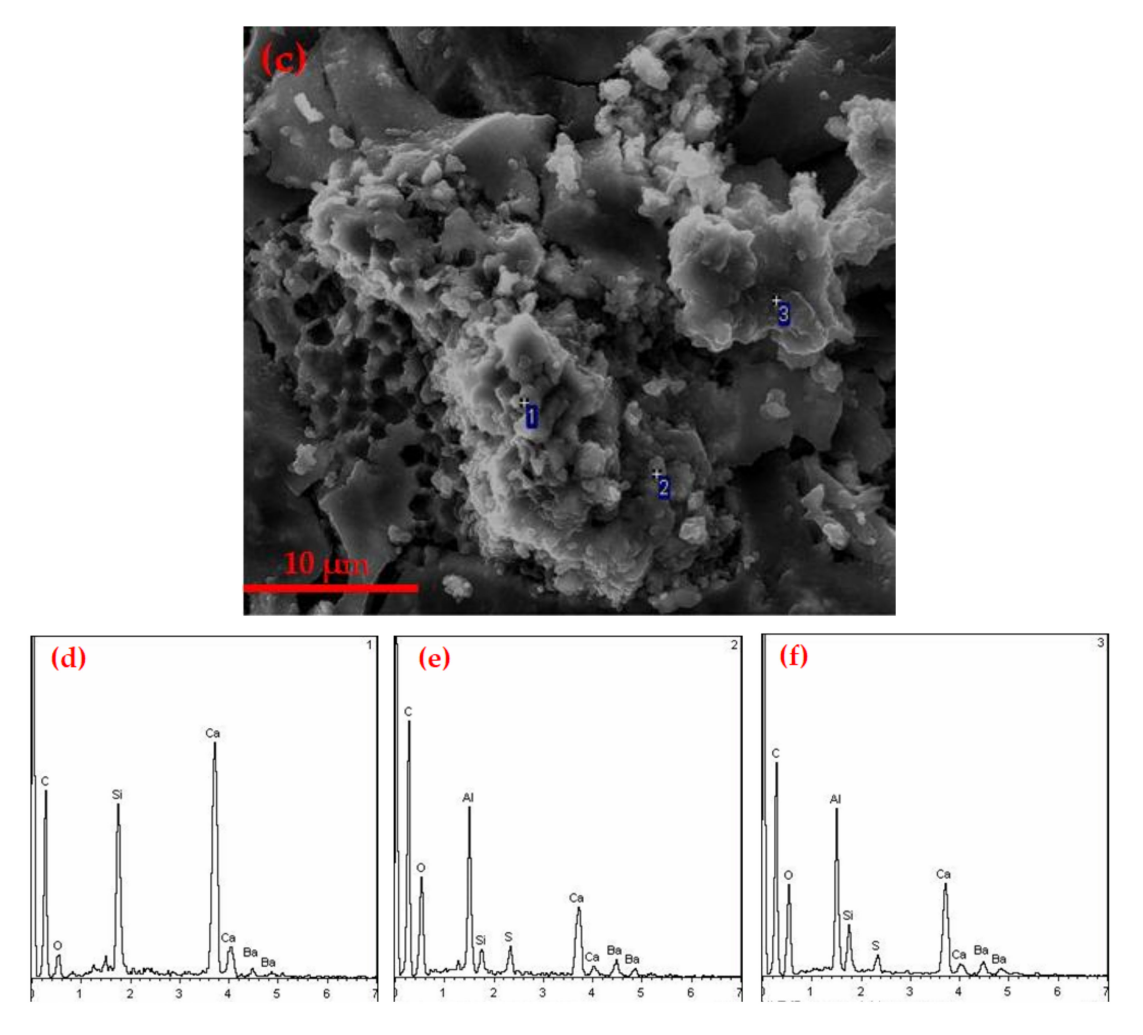
SEM of the (**a**–**c**) different C_4_AF contents of the clinker hydrated at 3 days, (**d**) 1-EDS, (**e**) 2-EDS, (**f**) 3-EDS.

**Figure 11:**
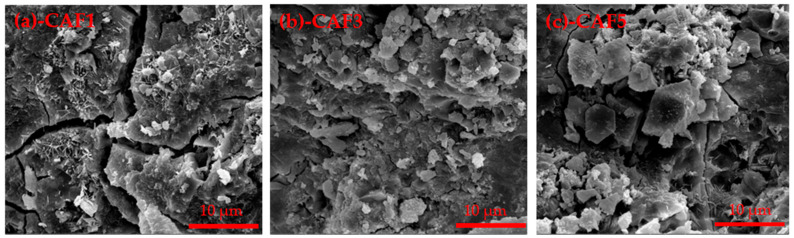
SEM of the different C_4_AF contents of the clinker hydrated at 3 days, (**a**)-CAF1, (**b**)-CAF3, (**c**)-CAF5.

The SEM images of the hydrated samples at different curing time sof CAF1, CAF3 and CAF5 are shown in Figure 10 and Figure 11. It can be seen from Figure 10 that 1-point was mainly unhydrated C_2_S, and 2 and 3-points were hydrated barium calcium sulfoaluminate with the flocculation shape. From the SEM image of CAF5, it can be seen that most of the products were colloidal substances, which were a mixture of AH_3_, BaSO_4_ and C–S–H gels.

Combined with an XRD and SEM analysis, it was believed that the hydration process of the cement was roughly as follows:

$$\begin{array}{l} C_{2.75}B_{1.25}A_{3}\bar{S}+H_{2}O\;\to \;{\mathrm{BaSO}}_{4}+{\mathrm{CAH}}_{10}+{\mathrm{AH}}_{3}\\ C_{2}S+H_{2}O\;\to \;C–S–H+\mathrm{CH} \end{array}$$

After C_2.75_B_1.25_A_3_$\bar{S}$ was contacted with water, BaSO_4_ was formed firstly, and the remaining part combined with H_2_O and formed CAH_10_ and AH_3_. It also can be seen from the SEM that a large amount of colloid covered around the CAH_10_ and the unhydrated clinker. It was found that some microcracks are common features of the different samples. This is a very common experimental phenomenon. Therefore, from the XRD patterns, the diffraction peak of C_2.75_B_1.25_A_3_$\bar{S}$ still appeared.

In summary, we believe that the main hydration products were BaSO_4_, CAH_10_, AH_3_ and C–S–H gels. The colloidal substance was filled in the crystal framework and the structure was dense. Therefore, the cement had high compressive strength.

### 3.3. Effect of C_2_F Content on Barium Calcium Sulfoaluminate Cement

#### 3.3.1. Mechanical Properties

**Figure 12:**
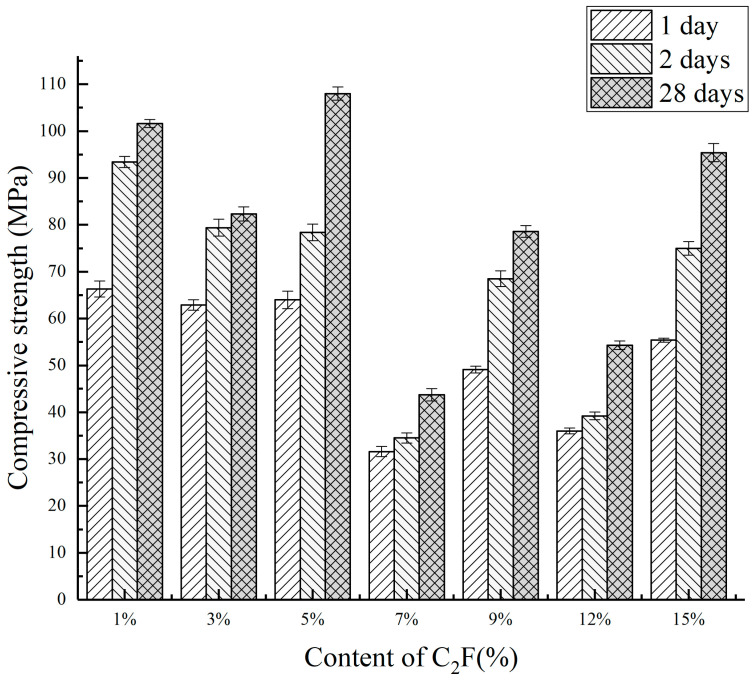
Compressive strength of the clinker at 1350 °C.

The change of compressive strength at each curing age is shown in Figure 12 and Table 3. It can be seen from Figure 12 that the compressive strength of the clinker fluctuated greatly. In the range of 1–5%, with the increased C_2_F content, the compressive strength was not changed significantly, and the compressive strength was better. When the C_2_F content was more than 7%, the compressive strength showed a downward trend. This may be caused by calcination at 1350 °C. The eutectic point of the system was lowered, and the minerals of the system were decomposed.

#### 3.3.2. XRD and SEM Analysis of Clinker

**Figure 13:**
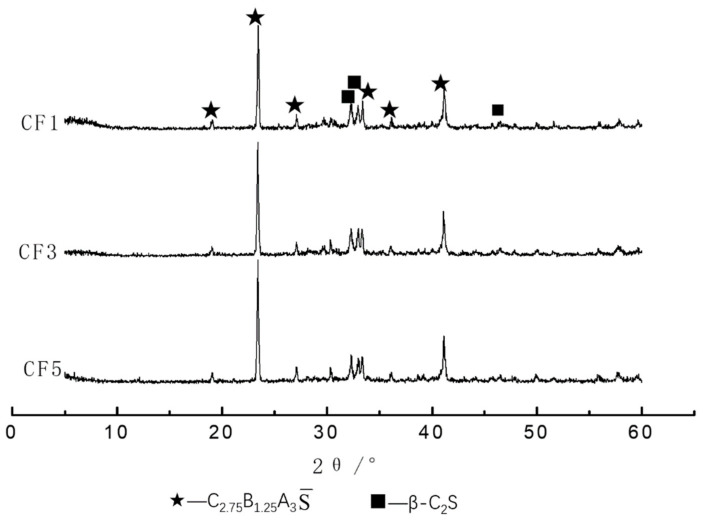
XRD patterns of different samples.

The X-ray diffraction analysis of the CF1, CF3 and CF5 clinkers was carried out with the D8-ADVANCE X-ray diffractometer (XRD), and the results are shown in Figure 13. It can be seen that the main minerals of the barium calcium sulfoaluminate cement clinker were C_2.75_B_1.25_A_3_$\bar{S}$ and β-C_2_S, and there was still a certain content of iron phase in the clinker, but the content was lower. The diffraction peak coincided with the diffraction peak of C_2_S, so it was not easy to observe.

**Figure 14:**
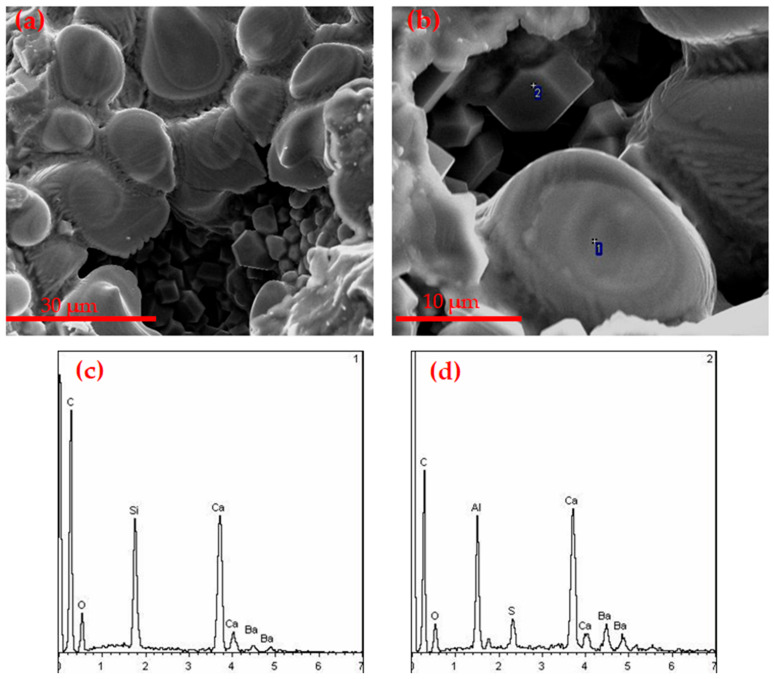
(**a**,**b**) SEM-EDS of CF3 clinker at 1350 ℃, (**c**) 1-EDS, (**d**) 2-EDS.

Figure 14 is the SEM-EDS image of the CF3 clinker mineral at 1350 °C. From Figure 14a, there were a lot of small particles resembling a hexagonal plate-shaped rhombic dodecahedron, the particle size was uniform and the grain boundary was clear. From the SEM-EDS at point “1” in Figure 14b, it can be seen that the mineral at this point was barium calcium sulfoaluminate, and the size of the mineral was about 5 µm. At the same time, there were a lot of small egg-like particles observed; the SEM-EDS at point “2” in Figure 14b showed that the soft-grained clinker mineral was β-C_2_S with a size of more than 10 µm. It was observed that the periphery of the small particles was surrounded by the liquid phase. Combined with the XRD analysis in Figure 13, it can be determined that the barium calcium sulfoaluminate mineral was well developed.

#### 3.3.3. XRD and SEM Analysis of Hydrated Samples

**Figure 15:**
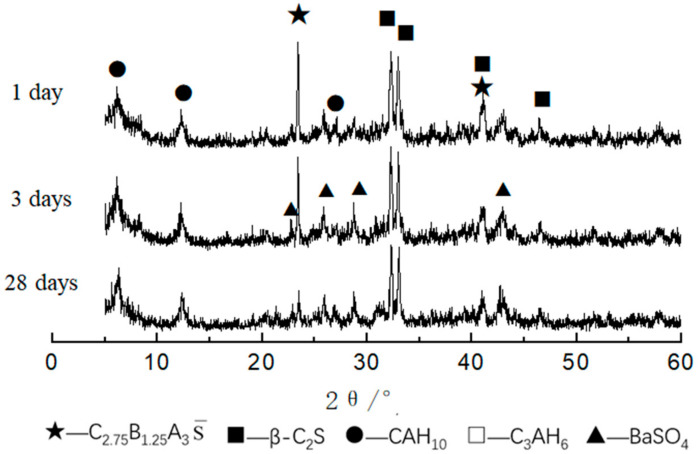
XRD patterns of sample CF1 at different hydrated days.

The results of the XRD analysis of the hydration products of the CF1 clinker at various ages are shown in Figure 15. It can be seen that the hydration rate of the barium calcium sulfoaluminate mineral was very fast, and the hydration products were mainly BaSO_4_, CAH_10_ and C_3_AH_6_. In addition, there were some unhydrated C_2.75_B_1.25_A_3_$\bar{S}$, and the unhydrated C_2.75_B_1.25_A_3_$\bar{S}$ diffraction peaks were relatively higher at 1 d and 3 d. The diffraction peaks of CAH_10_ gradually increased from 1, 3 and 28 days.

**Figure 16:**
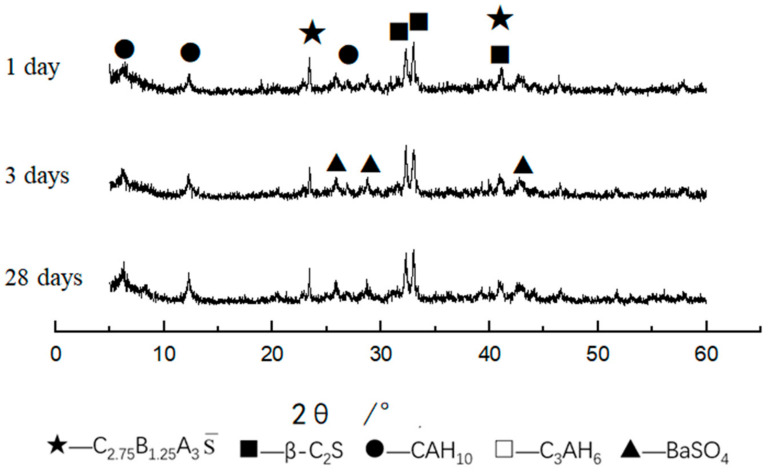
XRD patterns of sample CF3 at different hydrated days.

Figure 16 shows the XRD patterns of CF3 at different curing ages. It can be seen that the main hydration products were BaSO_4_, CAH_10_ and C–S–H gels. It can be seen that the diffraction peak of C_2.75_B_1.25_A_3_$\bar{S}$ decreased with the increased curing time. The diffraction peak of β-C_2_S decreased with the increased curing time, but the decreased trend was not obvious, indicating that the hydration speed of β-C_2_S was relatively slow. With the increased hydration time, the characteristic peak of CAH_10_ increased from 1 day to 28 days.

**Figure 17:**
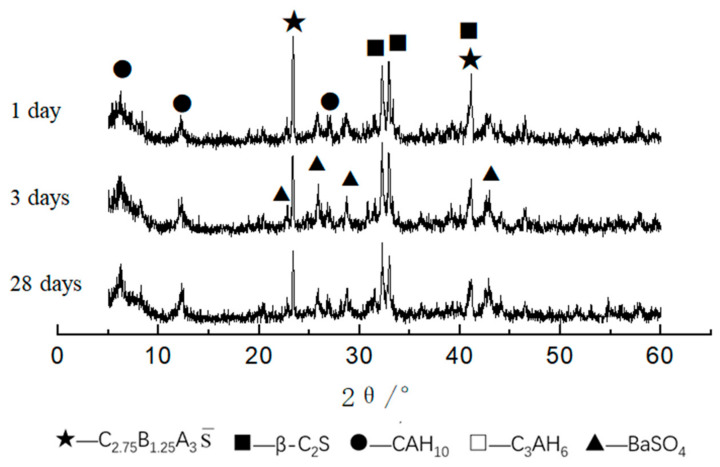
XRD patterns of sample CF5 at different hydrated days.

Figure 17 shows the XRD patterns of CF5 hydrated samples at different curing ages. It can be seen that the main hydration products were BaSO_4_, CAH_10_ and C_3_AH_6_. The diffraction peak of CAH_10_ from 1 day to 28 days increased. In addition, there were some unhydrated C_2.75_B_1.25_A_3_$\bar{S}$. The diffraction peak containing C_2.75_B_1.25_A_3_$\bar{S}$ decreased rapidly with the increased curing time. The diffraction peak of β-C_2_S decreased with the increased curing time, but the degree of decrease was not obvious, indicating that the hydration speed of β-C_2_S was relatively slow.

**Figure 18:**
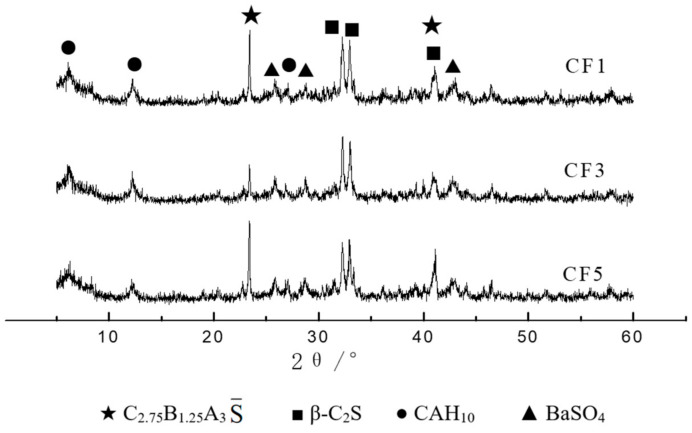
XRD of different samples at hydrated 1 day.

**Figure 19:**
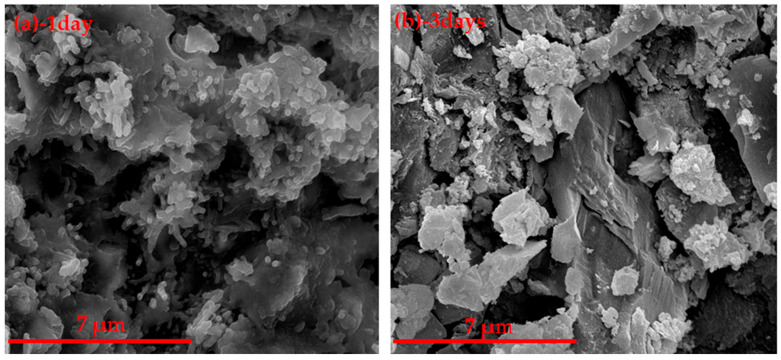
SEM of CF3 hydration paste, (**a**) 1 day, (**b**) 3 days.

It can be seen from Figure 18 that the main hydration products were BaSO_4_, CAH_10_, the unhydrated barium calcium sulfoaluminate and β-C_2_S. As shown in Figure 19, it can be seen that the hydration product after curing for 1 day was flocculent hydrated barium calcium sulfoaluminate, the structure was dense and the grain boundary of the cement became blurred, which explained the reason why the CF3 sample after curing for 1 d had the higher strength. After curing for 3 days, there were a lot of hydration products, which were hexagonal plates. The hydration products greatly increased and filled the pores, and the density of the cement paste increased. These were the reasons for the obvious increase in the 3 days compressive strength of the cement paste.

Combined with the XRD and SEM analysis, it is believed that the hydration process of the cement was roughly as follows:

$$C_{2.75}B_{1.25}A_{3}\bar{S}{+H}_{2}O\;\to {\;\mathrm{BaSO}}_{4}{+\mathrm{CAH}}_{10}{+\mathrm{AH}}_{3}$$

$$C_{2}{S+H}_{2}O\;\to \;C–S–H+\mathrm{CH}$$

After C_2.75_B_1.25_A_3_$\bar{S}$ reacted with water, BaSO_4_, CAH_10_ and AH_3_ were formed. It can also be seen from SEM that a large amount of colloid covered the hydrated product CAH_10_ and the unhydrated clinker. Therefore, the diffraction peak of C_2.75_B_1.__25_A_3_$\bar{S}$ (2θ = 24°and 41°) was still detected in XRD. In summary, we believe that the main hydration products were BaSO_4_, CAH_10_, AH_3_ and C–S–H gels. The structure was dense and the grain boundary of the cement became blurred. After curing for 3 days, the hydration products greatly increased and filled the pores, and the density of the cement paste was increased. The colloidal substance was filled in the crystal framework and the structure was dense. Therefore, the cement had high compressive strength.

## 4. Conclusions

This paper uses chemical reagents to burn calcium barium sulfoaluminate under laboratory conditions. The effects of different iron phases on the calcination and properties of barium calcium sulfoaluminate cement were studied. Combined with the XRD and the SEM-EDS analysis, the microstructure and composition of the barium calcium sulfoaluminate cement clinker and hydration samples were analyzed and characterized, and the following conclusions were drawn.

When the content of the iron phase increased, the compressive strength was decreased. While, when the content of C_4_AF was 15%, the samples exhibited better properties. The compressive strengths after curing for 1, 3 and 28 days were 74.3 MPa, 76.2 MPa and 102.5 MPa, respectively. A proper amount of the iron phase can reduce the eutectic point of the sintered mature material system, increase the amount of liquid phase, reduce the viscosity of the liquid phase, effectively accelerate the migration of mineral ions and promote the formation and growth of minerals. When the iron phase composition was C_4_AF and the iron phase content was 5%, the early hydration and later strength were better and the compressive strengths after curing for 1, 3 and 28 days were 73.2 MPa, 97.9 MPa and 106.9 MPa, respectively.

The composition and morphology of the mineral can be seen from the SEM-EDS. The oval-shaped particles were C_2_S minerals, the hexagonal plate-shaped or rhombohedral dodecahedral particles were barium calcium sulfoaluminate minerals and the iron phase was filled in between the two as solvent minerals. When the content of the iron phase was low, the liquid phase was low and the grain boundaries between particles were blurred, which was not conducive to the growth of minerals; when the content of the iron phase was higher, the eutectic point of the system decreased, and the clinker minerals melted at the same calcination temperature. A proper amount of iron phase was beneficial for the formation and growth of clinker minerals, the crystals were well developed and the grain boundaries were clear.

## Figures and Tables

**Table 1:** Mineral composition of clinkers.

Number	The Content of Mineral Composition/%		
	C_2.75_Ba_1.25_A_3_$\bar{S}$	C_2_S	C_4_AF
CAF0	60	40	0
CAF1	60	39	1
CAF2	60	37	3
CAF3	60	35	5
CAF4	60	33	7
CAF5	60	31	9
CAF6	60	28	12
CAF7	60	25	15

**Table 2:** Compressive strength of clinkers (MPa).

Number	1 Day	3 Days	28 Days
CAF0	65.5	71.2	96.5
CAF1	77.2	87.4	105.6
CAF2	68.4	67.5	64.5
CAF3	73.2	97.9	106.9
CAF4	70.8	66.5	100.6
CAF5	71.4	87.6	93.1
CAF6	70.2	72.3	98.2
CAF7	74.3	76.2	102.5

**Table 3:** Compressive strength of clinker at 1350 °C.

Number	The Content of Mineral Composition/%			Compressive Strength/Mpa		
	C_2.75_Ba_1.25_A_3_$\bar{S}$	C_2_S	C_2_F	1 Day	3 Days	28 Days
CF1	60	39	1	66.3	93.4	101.6
CF2	60	37	3	62.9	79.4	82.3
CF3	60	35	5	64.0	78.4	108.0
CF4	60	33	7	31.6	34.5	43.7
CF5	60	31	9	49.1	68.5	78.6
CF6	60	28	12	36.0	39.2	54.3
CF7	60	25	15	55.4	75.0	95.4

## Data Availability

The data presented in this study are available on request from the corresponding author.

## References

[1] Cheng X., Ye Z.M., Chang J., Lu L.C. (2009). Repairing bridges in coastal area with Ba bearing sulfoaluminate cement. Key Eng. Mater..

[2] Huang Y., Wang S., Hou P., Chen Y., Gong C., Lu L. (2014). Mechanisms and kinetics of the decomposition of calcium barium sulfoaluminate. J. Therm. Anal. Calorim..

[3] Yu J.C., Qian J.S., Tang J.Y., Fan Y.R. (2019). Effect of ettringite seed crystals on the properties of calcium sulfoaluminate cement. Constr. Build. Mater..

[4] Chenchen G., Jibao X., Shoude W., Lingchao L. (2016). Hydrating characteristics of modified Portland with Ba-bearing sulfoaluminate minerals. Ceram. Silik..

[5] Liu B., Wang S., Chen Y., Gong C., Lu L. (2016). Effect of waste gypsum on the setting and early mechanical properties of belite-C_2.75_B_1.25_A_3_$ cement. J. Therm. Anal. Calorim..

[6] Chang J., Xin C. (2001). Influence of fluorite on the Ba-bearing sulfoaluminate cement. J. Shandong Inst. Build. Mater..

[7] Wang Q. (2005). Inorganic non-metallic materials technology.

[8] Zhu M. (1996). Experimental Research on Iron Phase Formation and Hydration. Cem. Eng..

[9] Wang Y.M. (1996). Sulfoaluminate cement.

[10] Guo Y. (1988). Study on the formation mechanism of iron phases in ferro-aluminate cement. J. Chin. Ceram. Soc..

[11] Huang Y. (2013). Study on isothermal formation dynamics of calcium barium sulphaoluminate mineral. J. Inorg. Organomet. Polym. Mater..

[12] Li Y.J. (2000). Study on the effect of Fe_2_O_3_ on the formation of alite sulfoaluminate cement clinker mineral. Cement.

[13] Cheng X., Chang J. (2000). Study of Ba-bearing calcium sulfoaluminate minerals and cement. Cem. Concr. Res..

[14] Cheng X., Chang J. (2004). Study on the hydration of Ba-bearing calcium sulfoaluminate in the presence of gypsum. Cem. Concr. Res..

[15] Wang S., Huang Y., Gong C., Fu X., Lu L. (2016). Formation and early hydration characteristics of C_2.75_B_1.25_A_3_$ in binary system of C_2.75_B_1.25_A_3_$-C_2_S. Constr. Mater..

[16] Wang S., Huang Y., Gong C., Lu L., Cheng X. (2014). Formation mechanism of barium calcium sulfoaluminate mineral. Adv. Cem. Res..

[17] Zhang G. (2017). Effect of sulfoaluminate cement on the strength and water stability of magnesium potassium phosphate cement. Constr. Build. Mater..

[18] García-Maté M., Londono-Zuluaga D., de la Torre A.G., Losilla E.R., Cabeza A., Aranda M.A.G., Santacruz I. (2016). Tailored setting times with high compressive strengths in bassanite calcium sulfoaluminate eco-cements. Cem. Concr. Compos..

[19] Hargis C.W. (2013). Early age hydration of calcium sulfoaluminate in the presence of gypsum and varying amounts of calcium hydroxide. Cem. Concr. Compos..

[20] Garcia-Mate M. (2012). Rheological and hydration characterization of calcium sullfoaluminate cement pastes. Cem. Concr. Compos..

[21] Hargis C.W. (2014). Calcium sulfoaluminate hydration in the presence of gypsum, calcite, and vaterite. Cem. Concr. Compos..

[22] Zhao J., Chang J. (2017). Kinetic Analysis for Formation Process of Sr-Bearing Ye’elimite. J. Inorg. Organomet. Polym. Mater..

[23] Chang J., Li J., Han J., Zhang T. (2019). Traces of CH in a C_4_A_3_$-C_2_S hydration system. Constr. Build. Mater..

[24] Li X., Zhang Y., Shen X., Wang Q., Pan Z. (2014). Kinetics of calcium sulfoaluminate formation from tricalcium aluminate, calcium sulfate and calcium oxide. Cem. Concr. Res..

[25] Chang J., Zhang Y., Shang X., Zhao J., Yu X. (2017). Effects of amorphous AH_3_ phase on mechanical properties and hydration process of C_4_A_3_S¯-CS¯H_2_-CH-H_2_O system. Constr. Build. Mater..

[26] Cuesta A., de la Torre A.G., Losilla E.R., Peterson V.K., Rejmak P., Ayuela A., Frontera C., Aranda M.A. (2013). ChemInform Abstract: Structure, Atomistic Simulations, and Phase Transition of Stoichiometric Yeelimite. ChemInform.

[27] Kurokawa D., Takeda S., Colas M., Asaka T., Thomas P., Fukuda K. (2014). Phase transformation of Ca_4_[Al_6_O_12_]SO_4_ and its disordered crystal structure at 1073K. J. Solid State Chem..

[28] Feng X. (1996). The structure and quantum chemistry studies of 3CaO·3Al_2_O_3_· SrSO_4_. Cem. Concr. Res..

[29] Chang J., Shang X., Zhao J. (2015). Study on Sintering System of Calcium Barium Sulfoaluminate by XRD Quantitative Analysis. Appl. Sci..

[30] Álvarez-Pinazo G., Cuesta A., García-Maté M., Santacruz I., Losilla E.R., de la Torre A.G., León-Reina L., Aranda M.A.G. (2012). Rietveld quantitative phase analysis of Yeelimite-containing cements. Cem. Concr. Res..

[31] Cuesta A., Álvarez-Pinazo G., Sanfélix S.G., Peral I., Aranda M.A.G., de la Torre A.G. (2014). Hydration mechanisms of two polymorphs of synthetic ye’elimite. Cem. Concr. Res..

